# The Role of Gut Mucins in the Etiology of Depression

**DOI:** 10.3389/fnbeh.2020.592388

**Published:** 2020-11-05

**Authors:** Courtney Rivet-Noor, Alban Gaultier

**Affiliations:** ^1^Center for Brain Immunology and Glia, University of Virginia, Charlottesville, VA, United States; ^2^Department of Neuroscience, School of Medicine, University of Virginia, Charlottesville, VA, United States

**Keywords:** depression-epidemiology, microbiome, gut, mucus, metabolite, short chain fatty acid (SCFA), mucin

## Abstract

Major depressive disorders are global health problems that affect more than 6% of the U.S. population. Despite years of research, the etiology of depression remains unclear. Historically, it was believed that depression started within the central nervous system (CNS), but alternative hypotheses have recently challenged this dogma. Indeed, experimental and clinical evidence show that the gut microbiome could be an active player in depression initiation. The composition of bacterial species in depressed patients is significantly different from control microbiomes, and the transfer of the microbiome from depressed patients is sufficient to initiate depressive symptoms in animals. Additionally, the gut microbiome is known to change in the presence of depression risk factors such as chronic stress. While there is strong evidence delineating a role for microbial dysbiosis in depression, the initiating event for this dysbiosis remains unknown. Within the gut, microbiota reside in the mucus layer, a critical gel-like barrier involved in protecting the host from unwanted pathogen interactions, as well as regulating the immune system. Though the mucus layer is often ignored in the face of dysbiosis, it represents a dynamic and important piece of host machinery that has the potential to impact a wide variety of biological processes. Here, we review evidence supporting the novel concept that stress can modify the delicate mucus-microbiome balance, initiating dysbiosis, and ultimately leading to depression.

## Introduction and History of Depression

Depression is one of the most prevalent disorders worldwide, impacting over 16% of the world’s population (Dean and Keshavan, 2017). It is estimated to cost society over $200 billion annually (Dean and Keshavan, 2017). Much of the understanding of this disorder has come from the accidental discovery of monoamine altering drugs, as well as large-scale genetic studies (Loomer et al., 1957; Levinson, 2006). Both the use of monoamine altering drugs and genetic studies have implicated the serotonergic, adrenergic, and dopaminergic pathways—as well as neurotrophic agents such as brain derived-neuroprotective protein—in the pathology of depression (Loomer et al., 1957; Levinson, 2006). As these studies implicate neurotransmitters and other proteins involved in the central nervous system (CNS) function, therapeutic development has centered around neurotransmitters, neurogenesis, and neuronal plasticity. However, the hypotheses underlying these research avenues cannot explain all of the observations in patients being treated with antidepressants. For example, it can take months to see mood improvements from treatment with serotonin reuptake inhibitors (SSRIs), even though neurotransmitter availability is corrected within the first hours of drug administration (Anderson, 2004). Similarly, ketamine, which is believed to correct depressive symptoms by increasing neurogenesis and dendritic spine formation, can act within hours of infusion—before changes in neuronal plasticity have been observed (Soumier et al., 2016). While many people have been treated successfully with these methods, as many as 30% of patients diagnosed with depression remain resistant to treatment (Fitzgerald et al., 2020). Furthermore, as the heritability of depression is only thought to account for 40–50% of cases, environmental factors that influence mood disorders must be considered as potential therapeutic targets (Levinson, 2006). In light of this, researchers have started exploring additional contributors to depression that lie outside of dysregulated neuronal circuitry—those that are impacted by the environment. One particular area of interest is the gut-brain axis.

## The Gut-Brain Axis in Depression

There is significant foundational evidence supporting the involvement of the gut-brain axis in mood disorders. For example, people diagnosed with inflammatory bowel disease are at significantly higher risk for depression (Byrne et al., 2017). While the mechanistic link between intestinal problems and increased risk for depression remains unclear, the microbiome offers a potential mechanism through which the gut can influence depression. The intestinal microbiome is composed of trillions of microorganisms that execute vital functions for the host. Numerous studies have demonstrated that the gut microbiome has a profound impact on the CNS during both homeostatic and pathological conditions (Durack and Lynch, 2019). The pathological contribution of the microbiome to CNS disorders is primarily attributed to altered communication between the gut and brain, mediated by a state of microbial dysbiosis. This has become particularly evident in depression, where the microbiomes of depressed patients are markedly different than controls (Naseribafrouei et al., 2014; Jiang et al., 2015). In these studies, a general increase in the phyla of *Bacteroidetes* was overserved in depressed patients (Naseribafrouei et al., 2014; Jiang et al., 2015). Additionally, an underrepresentation of Lachnospiraceae at the family level and *Bifidobacterium* and *Lactobacillus* at the genus level has been described in depressed cohorts (Naseribafrouei et al., 2014; Jiang et al., 2015; Aizawa et al., 2016). Interestingly, probiotics are effective in reducing depressive symptoms and microbiome transfers from depressed human patients into rats were sufficient to induce depressive-like behaviors in the animals, further supporting the idea that the microbiome can initiate depression (Kelly et al., 2016; Rojas et al., 2019). However, gaps remain in our understanding of how the gut-brain axis contributes to depression. Efforts must be made to: (1) understand the routes through which the microbiome initiates depression; and (2) discover the factors that initiate microbiome dysbiosis.

## Stress and the Microbiome

Stress is a major risk factor for depression (Hammen, 2005). While chronic stress and the associated production of glucocorticoids can inhibit immune system function (Morey et al., 2015), they can also affect other aspects of physiology (Thau et al., 2020). Stress has also been associated with many types of intestinal distress, one of which includes increased gut permeability (Kelly et al., 2015). This stress-associated change in the gut epithelium allows for increased levels of pro-inflammatory cytokines (Bailey et al., 2011), which play an important role in propagating depressive behaviors (Dantzer et al., 2008; Fritz et al., 2016, 2018). Additionally, stress has been shown to change the composition of the microbiome—altering metabolites and the immune system in ways that feedback on depression (Marin et al., 2017). For example, mice exposed to chronic mild stress (CMS) exhibit depressive-like behaviors and a reduction in *Lactobacillus* (*Lacto*, Marin et al., 2017). Importantly, when these mice are supplemented with *Lacto* during CMS, they no longer exhibit depressive behaviors (Marin et al., 2017). Taken together, this indicates that an initiating event in depression could be through stress-induced microbial dysbiosis. Given the correlation between stress-induced dysbiosis, modified gut permeability, and depressive outcomes, it would appear logical that these events are linked. As such, research has begun to examine how the microbiome may be therapeutically altered in depression to restore gut homeostasis. However, the exact mechanism through which stress initiates a change in the microbiome that results in depression remains unclear. Thus, it is critical that current research into the mechanisms of depression focus on factors at the intersection of stress, the microbiome, and gut permeability.

## Mucins and the Microbiome

Mucus is a gel-like layer made of highly glycosylated proteins called mucins, which coats many epitheliums within the body. Mucins come in two distinct types: soluble and membrane-bound. Soluble mucins compose the gel-like structure that coats an organ, while membrane-bound mucins remain attached to the cell epithelium and make up the glycocalyx (Johansson et al., 2011). One of the functions of mucus is to act as a protective barrier for an organ or tissue, preventing unwanted pathogen interactions (Kebouchi et al., 2020). This is especially critical in the gut, where trillions of microbes have the potential to trigger the host immune system and cause disease (Schroeder, 2019). In addition to this protective function, the gut mucus layer also serves as a nutrient reservoir and anchor point for commensal bacteria (Johansson et al., 2011). While most mucins have unknown functions, some have been found to have roles in the immune system, cancer, irritable bowel syndrome, and colitis (Van der Sluis et al., 2006; Kebouchi et al., 2020).

**Figure 1:**
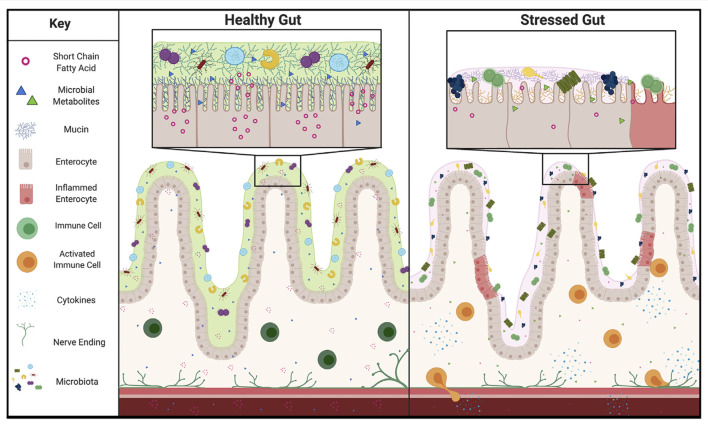
Proposed representation of Healthy vs. Stressed Gut. In the healthy gut a thick mucosal layer keeps microbiota separated from the epithelium. These species produce many short chain fatty acids and other metabolites that can act on the rest of the body through several routes, including: the immune system, blood stream, or vagal nerve. In the stressed gut the mucosal layer composition is markedly different. A thinner mucosal layer and different composition of proteins selects for a different subset of microbial species and brings those species closer to the epithelium. This change in distance allows for inflammation of the gut lining and activation of the immune system. In addition, the change in microbiome composition alters the amount of short chain fatty acids and presence of other metabolites produced, altering homeostasis and feeding back on depressive behaviors.

The mucosal layer is uniquely positioned to shape the microbiome. As the natural food source and anchor point for the microbes that live in the gut, the mucosal layer serves as a connection point for the microbiome (Johansson et al., 2011). Although microbes can influence the mucus layer, mucins can also impact the content of the microbiome and alter factors that are responsible for communication between the gut and the brain. Indeed, the mucosal layer can select for specific bacteria based on patterns of mucin glycosylation in the gastrointestinal (GI) tract (Schroeder, 2019). It has also been demonstrated that stress, a major risk factor for depression, can shift the O-glycosylation patterns of mucins in rats (Silva et al., 2014), supporting the idea that stress may be able to change the mucosal layer in a way that induces dysbiosis. Additionally, glycocalyx mucins have been shown to influence the shape of microvilli, critical structures of the intestine involved in nutrient absorption, and epithelial and microbiome interactions (David et al., 2012; Shurer et al., 2019). Increased oxidative stress and inflammation, also associated with chronic stress, have been shown to change microvilli structures, further suggesting that stress could tune the microbiome composition (Vanitallie, 2002). Strikingly, two genome-wide association studies have implicated mucins in depression (Dunn et al., 2018; McClain et al., 2020). In a 2020 study examining genetic mutations in treatment-refractory depression, pathway analysis revealed significant enrichment in the O-linked glycosylation pathway of mucins (McClain et al., 2020). Additionally, mucin 13 has been specifically implicated in a genome-wide association study of depressive symptoms in a Hispanic community (Ware et al., 2015; Dunn et al., 2018). Taken together, this suggests that stress induced mucus changes may be responsible for microbiota dysbiosis. This induction of dysbiosis could then continue to feedback on depression through previously described contributors to depression, such as microbial metabolites or the immune system (Figure 1).

## Mucins and Gut Microbiome Metabolites

The gut microbiome produces essential metabolites, such as neurotransmitters, tryptophan metabolites, and short-chain fatty acids (SCFAs), which can act on the CNS (Morris et al., 2017). Under a state of dysbiosis, induced through mucin changes, gut-derived molecule ratios may change, contributing to the etiology of depression (Carding et al., 2015; Morris et al., 2017). SCFAs are a group of metabolites produced by anaerobic fermentation of indigestible dietary carbohydrates and can act in the body in a multitude of ways (Caspani et al., 2019). SCFAs have been shown to inhibit histone deacetylase (HDAC), an enzyme that can change the epigenetic landscape and has been shown to increase depressive-like behaviors in mice (Gundersen and Blendy, 2009). SCFAs are also believed to influence the CNS through vagal nerve fibers. Activation of the vagus nerve by SCFAs is believed to reduce appetite and the reward responses to food, both known symptoms in depression patients (Goswami et al., 2018). In addition to the consequences of large scale dysbiosis, mucins are directly processed into SCFAs, suggesting that even if overall gut homeostasis is retained, a change in the mucosal layer could still modulate SCFA availability and feedback on depression (Kim, 2018).

Dysbiosis caused by stress-induced mucosal composition changes can also influence mood disorders through tryptophan derivatives (Marin et al., 2017). Tryptophan is an essential amino acid that must be obtained from the diet. Once available, tryptophan can be metabolized through multiple pathways to produce various biologically active molecules (Gheorghe et al., 2019). The gut microbiome is closely connected with tryptophan metabolism, as mice lacking a microbiome have been shown to have reductions in its downstream metabolites (Gheorghe et al., 2019). Additionally, a reduction in tryptophan metabolites and the enzymes associated with its metabolic pathways, such as indoleamine-2,3-dioxygenase, are reduced in stress-induced depression and correlate with reduced expression of *Lactobacillus* (Marin et al., 2017). It is believed that the availability and ratio of these molecules are critical for mediating depression through the availability of the neurotransmitter serotonin, the tryptophan metabolite kynurenine, and the presence of downstream, neurotoxic tryptophan derivatives 3-hydroxykynurenine and quinolinic acid (Savitz, 2017). The presence of appropriately glycosylated mucins can also be specifically tied to tryptophan derivatives. Bacterial species have been shown to utilize mucin glycans as a food source to metabolize tryptophan, highlighting another way mucin availability may influence the tryptophan pathway and feedback on depressive behaviors (Wlodarska et al., 2017).

Last, dysbiosis can alter the production of neurotransmitters in the gut (Caspani et al., 2019). This alteration in neurotransmitter availability, driven by mucosal changes, can feedback on more traditional aspects of depression. For example, dysbiosis is associated with serotonin transporter deficiency, a known correlate with depression (Singhal et al., 2019). While changes in the mucosal layer could impact microbial metabolites that have been associated with depression, changes in this protective layer can also impact another major system that is closely linked to depression: the immune system.

## Mucins and the Immune System

In addition to impacting bacterial metabolites, changes in the mucosal layer could also influence the activation of the immune system. As the mucosal layer is the first line of defense preventing unwanted pathogen interactions with the epithelial layer of the gut, it is a critical piece of host protection (Kebouchi et al., 2020). Accordingly, disruption of the mucus layer allows for spontaneous disease development, such as colitis and colorectal cancer, and induces sweeping microbiome changes (Van der Sluis et al., 2006; Wu et al., 2018). Many of the diseases associated with a deficient mucosal layer are propagated by a dysregulated immune system (Velcich et al., 2002; Van der Sluis et al., 2006). In these ailments, a disrupted mucus layer allows for bacterial species to migrate closer to the epithelium and trigger the immune system (Figure 1), resulting in the release of damaging inflammatory cytokines (Schroeder, 2019). As depression is also linked to an activated immune system, it is intriguing to consider that a change in the mucosal layer could evoke a similar effect (Dantzer et al., 2008; Fritz et al., 2016; Gheorghe et al., 2019).

Depression is correlated with an increase in markers of bacterial translocation, gut permeability, and toll-like receptor activation, all important measures of increased immune activity in the gut (Kéri et al., 2014). Additionally, cytokines shown to increase with changes in the microbiome and gut permeability are known to be elevated in depressed patients (Kéri et al., 2014; Schirmer et al., 2016; Kiecolt-Glaser et al., 2018). These cytokines can sensitize the cortisol response pathway, influence the kynurenine pathway, or act directly on neurons and/or glial cells within the brain to propagate depression symptoms (Dantzer et al., 2008; Fritz et al., 2016; Hoshino et al., 2017; Gheorghe et al., 2019). For example, pro-inflammatory cytokines have been shown to stimulate the release of corticotrophin release factors in the rat brain (Berkenbosch et al., 1987). Consequently, this increased level of circulating stress hormone desensitizes the HPA axis to negative feedback loops, allowing it to stay active for longer and prolonging the experience of stress, a known risk factor for depression (Hammen, 2005). Additionally, increased levels of stress hormones are associated with atrophy of brain areas important in depression- the hippocampus and prefrontal cortex (McEwen and Magarinos, 1997). Furthermore, increasing the presence of cytokines by bringing the microbiome in closer contact with the immune system can also influence the kynurenine pathway. In mice, proinflammatory cytokines have been shown to increase the amount of indoleamine 2,3-dioxygenase (IDO), an enzyme that converts tryptophan to kynurenine, by directly stimulating enzyme production (O’Connor et al., 2008). This increase in the production of kynurenine is correlated with depressive-symptoms and injection of this metabolite is sufficient to induce depressive-like behaviors in animals (O’Connor et al., 2008). Lastly, cytokines may be able to influence neurons directly. It has been shown that cytokines can decrease synaptic plasticity and cause damage to existing dendrites (Schirmer et al., 2016). While uncertainty about this in the context of depression remains, it is now appreciated that neurons express cytokine receptors and that cytokines can influence CNS function (Filiano et al., 2016).

Another avenue in which the immune system may be altered through changes in the mucosal layer is through Th17 cells. In depression, patients are thought to have a disproportionate number of pro-inflammatory Th17s compared to anti-inflammatory T regulatory (Treg) cells, leading to higher rates of inflammation (Han et al., 2015). Additionally, studies have reported increased levels of IL-17, the primary cytokine produced by Th17s, in patients with depression (Tsuboi et al., 2018). Interestingly, as Th17s are primarily responsible for helping control extracellular bacterial and fungal infections, they are found at a greater proportion in the gut. As the mucosal layer acts to separate the bacteria and fungi that are housed in the intestine, a change in this barrier would bring these foreign species closer to the ever-present Th17s and could cause activation. Intriguingly, it has been found that Th17s are elevated in the brain after stress exposure and that the adoptive transfer of Th17s induces depressive like-phenotypes in animals (Beurel et al., 2013). Furthermore, blocking IL-17 or RORγT, the master transcription factor for Th17s was found to prevent the onset of depressive behaviors (Beurel et al., 2013). While this work is still in its nascent stages, it represents a promising line of inquiry into how another gut-associated immune component plays a major role in depressive outcomes.

While each of these aspects of the immune system is not yet directly tied to changes in the complex array of mucins within the gut, they represent interesting points of connection. Both Th17s and pro-inflammatory cytokines are highly associated with depression and changes in gut immune activation. Further highlighting this connection, cytokines and Th17s are known to increase when the microbiome comes close to host epithelial cells (Kéri et al., 2014; Han et al., 2015). As one of the mucosal layer’s primary functions is to prevent the microbiome from coming in contact with host cells, it stands to reason that a change in this protective layer could bring microbes in close enough proximity to the immune system to cause activation. Theoretically, once activated, these gut-associated pro-inflammatory cytokines and Th17s would be able to travel throughout the body and act on the brain to induce depression in ways that have been previously detailed in the aforementioned depression studies (Dantzer et al., 2008; Beurel et al., 2013; Fritz et al., 2016; Gheorghe et al., 2019).

## Conclusions

Depression remains a significant public health concern. Research has discovered avenues through which depression symptoms may be propagated and therapeutics that attempt to circumvent these processes. However, depression treatments are effective in only a subset of patients and often do not correct all symptoms. In light of this, more work is needed to investigate the root causes of depression to provide alternative management options for the disease.

Because stress represents a major risk factor for depression, areas of the body that are impacted by stress should be looked at to identify potential depression initiating factors. The microbiome is heavily impacted by stress and changes to its composition are associated with depressive outcomes. However, therapeutic targeting of the microbiome remains difficult given the complexity of bacterial interactions and variability between patients. Additionally, how stress changes the microbiome is not understood. This knowledge gap may be the missing link capable of connecting many of the factors that propagate depressive states and provide a targetable option for future therapeutics. Logically, it follows that closer examination of what areas stress can act on near the microbial niche may provide clues to how it can cause dysbiosis. One such candidate lies in the mucosal layer of the small intestine.

Mucins are uniquely positioned to be influenced by stress and also shape the microbial niche. These properties allow them to serve as a potential site of initiation for stress-induced microbial dysbiosis that could feedback on depression (Figure 1). As this layer has been shown to select for specific bacterial species, change the structure of cellular membranes to influence microbial interactions, and respond to stress, it provides a potential mediator for stress-induced-microbiome changes. These microbiome changes could feedback on depressive states through several known routes including altering levels of SCFAs, neurotransmitters, and kynurenine metabolites. Furthermore, a change in mucins and the mucosal layer has the potential to influence the immune system. Serving as the main defense against host-microbe interaction, a change in mucus structure or composition would alter the distance between the microbiome and the host immune system. In the event the mucosal layer was altered by stress, the microbial species present in the gut could encroach upon the host epithelial layer and cause activation of the immune system. Supporting this idea, pro-inflammatory cytokines, and Th17s that are found in the gut after immune activation are also found to be elevated in depressed patients. While the origin of these immune molecules in depression is not known, their presence prompts interesting questions about the gut and observed depressive phenotypes.

In terms of long-term disease intervention, targeting the mucosal layer allows for a therapeutic opportunity that bypasses the direct manipulation of the microbiota, while potentially influencing the pathways that are affected by depression. Mucin therapeutics represent an area of treatment that has the potential to be broadly applicable across patients with differing microbiome compositions. Mucins represent an untouched area of research in the depression field, yet are poised to influence many important aspects of the disease. Further work is needed to uncover the full potential of the mucosal layer in depression and other microbial dysbiosis associated diseases, but a broader understanding of their influence has the potential to fundamentally alter our approach to depression therapeutics.

## Author Contributions

CR-N and AG conceptualized the format of this review. CR-N wrote and designed the figure. AG edited and guided refinement of the manuscript. All authors contributed to the article and approved the submitted version.

## Conflict of Interest

The authors declare that the research was conducted in the absence of any commercial or financial relationships that could be construed as a potential conflict of interest.
